# Comparative metagenomics of hydrocarbon and methane seeps of the Gulf of Mexico

**DOI:** 10.1038/s41598-017-16375-5

**Published:** 2017-11-22

**Authors:** Adrien Vigneron, Eric B. Alsop, Perrine Cruaud, Gwenaelle Philibert, Benjamin King, Leslie Baksmaty, David Lavallée, Bartholomeus P. Lomans, Nikos C. Kyrpides, Ian M. Head, Nicolas Tsesmetzis

**Affiliations:** 1 School of Civil Engineering and Geosciences, Newcastle University, Newcastle Upon Tyne, UK; 2 Shell International Exploration and Production Inc., Houston, Texas USA; 3 DOE Joint Genome Institute, Walnut Creek, California USA; 4 INRA, UMR1062 CBGP, F-34988 Montferrier-sur-Lez, France; 5 Shell Global Solutions International B.V., Rijswijk, Netherlands

## Abstract

Oil and gas percolate profusely through the sediments of the Gulf of Mexico, leading to numerous seeps at the seafloor, where complex microbial, and sometimes animal communities flourish. Sediments from three areas (two cold seeps with contrasting hydrocarbon composition and a site outside any area of active seepage) of the Gulf of Mexico were investigated and compared. Consistent with the existence of a seep microbiome, a distinct microbial community was observed in seep areas compared to sediment from outside areas of active seepage. The microbial community from sediments without any influence from hydrocarbon seepage was characterized by *Planctomycetes* and the metabolic potential was consistent with detrital marine snow degradation. By contrast, in seep samples with methane as the principal hydrocarbon, methane oxidation by abundant members of ANME-1 was likely the predominant process. Seep samples characterized by fluids containing both methane and complex hydrocarbons, were characterized by abundant *Chloroflexi* (*Anaerolinaceae*) and deltaproteobacterial lineages and exhibited potential for complex hydrocarbon degradation. These different metabolic capacities suggested that microorganisms in cold seeps can potentially rely on other processes beyond methane oxidation and that the hydrocarbon composition of the seep fluids may be a critical factor structuring the seafloor microbial community composition and function.

## Introduction

Gulf of Mexico sediments harbor numerous shallow sources of methane and other hydrocarbons. These include methane seeps^1,2^, hydrocarbon seeps^3–5^, gas hydrate mounds^6–8^, subsurface brines^9^, asphalts^10–12^ and mud volcanoes^13^. These seeps sustain conspicuous microbial communities identifiable on the seafloor by the presence of white or colored microbial mats, aggregates of vesicomyid clams and/or tubeworms depending on the fluid flow rate and the hydrogen sulfide concentrations^14^. Numerous studies have focused on the microbial communities present in these environments and a large diversity of microorganisms dependent on methane and sulfur cycling has been observed^15^.

Particular attention has been devoted to consortia of archaeal anaerobic methanotrophs (ANME) and sulfate-reducing bacteria (SRB) that couple the anaerobic oxidation of methane to sulfate reduction^15^. Although the mechanism of anaerobic methane oxidation (AOM) has been investigated since 2000^16^, and seems to involve a reverse methanogenesis pathway^17–19^, the exact process remains unclear and different variants may occur depending on the species involved^18,20^. Indeed, different groups of ANME (ANME-1a,-b, ANME-1Guaymas, ANME-2a, -2b,-2c, ANME-3, ANME-2d/GoM Arc I/AAA)^15,21–23^, closely affiliated to the *Methanosarcinales* and *Methanomicrobiales*, have been identified. In addition, sulfate-reducing *Deltaproteobacteria* (SEEP SRB1a of the *Desulfosarcina*/*Desulfococcus* group, SEEP SRB2, *Desulfofervidus* lineages and some members of the genus *Desulfobulbus*) have been identified in syntrophic methane-oxidizing consortia in these and other environments^24–28^. However ANME-1 and ANME-3 have been observed as monospecific aggregates questioning whether the association between ANME archaea and sulfate-reducing deltaproteobacteria is obligate^21,22,29^. AOM is widely considered to be the main process occurring in cold seep sediments due to the ubiquity of ANME sequences in 16S rRNA gene surveys and the microscopic detection of the striking aggregates of ANME and SRB^30^. Consequently, the intriguing microbial consortia involved in AOM have been the focus of the majority of the microbial investigations of marine seep sediments.

Other members of the microbial communities in these environments have been somewhat overlooked even though numerous uncultured archaeal and bacterial lineages co-occur with the ANME/SRB consortia^14^. The relative proportion of non-ANME/SRB lineages has been shown to increase with the distance from the seep source and the proximity of faunal assemblages^14^, suggesting the existence of ecological niches other than AOM in these seep sediments. Furthermore, the presence of oil and higher hydrocarbons in the percolating fluid is also suspected to play an important role in shaping the microbial communities present, by selecting for non-methane-hydrocarbon degraders, methanogens and acetogens^11^. Indeed, activity measurements in Gulf of Mexico seeps suggest that significant rates of sulfate reduction are supported by non-methane hydrocarbon degradation and that an uncultured group of *Deltaproteobacteria* may be responsible for degrading higher molecular weight hydrocarbons^11^. This has been confirmed by the detection of genes involved in anaerobic degradation of alkanes in sulfate-reducing bacteria^31^, successful cultivation of hydrocarbon degrading sulfate reducers from seep sediments^32,33^, and stable isotope probing experiments^34^. Furthermore, single cell genomics and assembly of genomic bins from large metagenomic datasets, has highlighted the metabolic potential of some of the microbial “dark matter” from marine sediments. These approaches have revealed the capacity for organic matter degradation in Marine Benthic Group D archaea, *Bathyarchaeota*, *Atribacteria* and *Dehaloccocoidia*, and potential for methanogenesis in *Bathyarchaeota* ^35–39^. However genome-centric metagenomics only provide single pieces of the puzzle and the overall picture of microbial community function in marine deep-sea ecosystems remains fragmented.

To better understand the community structure and different functions present in marine seep ecosystems, a comparative multigenic and metagenome sequencing approach was used on samples from three area of the Mississippi Canyon in the Gulf of Mexico. The Mississippi Canyon harbors numerous sea floor mounds with shallow methane and thermogenic gas hydrates^40^. Two cold seep sites with different hydrocarbon compositions, potentially reflecting methane and thermogenic gas hydrates and samples from outside any area of active seepage were compared. Common and unique metabolic features of the microbial communities were identified and complemented with geochemical data to provide new insights into the different microbial processes in marine hydrocarbon seep environments.

## Results

### Geochemical characterization of the Gulf of Mexico sediments

Push core sediment samples were collected in October 2015 at three different locations in the Mississippi Canyon of the Gulf of Mexico. Site 1 (PC5 and PC6) and Site 2 (PC9 and PC10), located 70 meters appart, presented unambiguous geochemical evidence of seepage and white mat-like traces at the sediment surface. Two push cores (PC11 and PC12) were also taken 200 meters away, at locations remote from any area of visible active seepage as ‘background’ reference samples. Different pore water compositions were observed in these sediment cores (Table 1). Nitrate was below the detection limit (<0.08 mM) in all samples. Sulfate was partially depleted, relative to seawater, throughout the Site 1 seeps (PC5 and PC6) and core PC10 from Site 2. In the PC10 core, 7.88 mM and 12.93 mM sulfate was present at 3–4 cmbf and 10–12 cmbsf respectively, whereas sulfate concentration decreased with depth in sediment cores from Site1 with 13 mM sulfate at 3–4 cmbsf decreasing to 5.3 mM at 11–12 cmbsf in PC5 (Table 1). By contrast, sediment cores from outside the seep area (PC11 and PC12) as well PC9 from Site 2 presented opposite contrasting profile with sulfate depletion at 3–4 cmbsf (10 mM sulfate) and seawater sulfate concentration (27 mM) at 10–12 cmbsf. Additionally thiosulfate was detected in all cores of the seep sites except in PC9 (Table 1). Overall, low methane concentrations were detected in the sediment samples with methane concentrations under 0.05 µM in all sediments samples from outside the seep area as well as in PC9. Methane at concentrations of 2.55 µM and 169 µM was detected at the bottom of PC10 (Site 2) and PC5 (Site 1) respectively (Table 1). Additionally, GC analysis of sediment from Site 1 pushcores (PC5 and PC6) showed the presence of an unresolved complex mixture (UCM) of hydrocarbons with various aromatics compounds and hopanoids (Table 1), as previously observed in gas hydrates of the Mississippi Canyon^40^. Methane was the only hydrocarbon detected in sediments from Site 2 (PC9 and PC10).

**Table 1:** Geochemical description and microbial abundance of the samples. UCM: Uncharacterized complex mixture. ND: Not Determined.

**Sampling Site**	Pushcore Name	Sediment Layer	Nitrate (mM)	Sulfate (mM)	Thiosulfate (mM)	Chloride (mM)	Methane (µM)	Oil	*Bacteria* (16S rRNA gene.g^−1^)	*Archaea* (16S rRNA gene.g^−1^)
Site 1 seeps	PC5	3–4 cmbsf	<0.08	13.72	0.08	418.3	0.27	UCM (aromatics and hopandoids)	8.14 ± 0.9 × 10^8^	1.59 ± 0.05 × 10^8^
		10–12 cmbsf	<0.08	5.31	0.7	551.88	169		1.52 ± 0.3 × 10^9^	1.62 ± 0.02 × 10^9^
	PC6	3–4 cmbsf	<0.08	12.36	0.06	409.87	<0.05	UCM (aromatics and hopandoids)	6.38 ± 0.6 × 10^8^	8.83 ± 2.2 × 10^7^
		10–12 cmbsf	<0.08	10.5	0.65	549.23	NA		6.24 ± 0.9 × 10^8^	5.65 ± 0.2 × 10^8^
Site 2 seeps	PC10	3–4 cmbsf	<0.08	7.88	0.11	434.91	0.06	ND	6.36 ± 0.9 × 10^8^	6.97 ± 0.6 × 10^8^
		10–12 cmbsf	<0.08	12.93	0.61	544.03	2.55		8.84 ± 1.1 × 10^8^	1.18 ± 0.05 × 10^9^
	PC9	3–4 cmbsf	<0.08	10.74	<0.03	405.69	<0.05	ND	3.26 ± 0.2 × 10^8^	5.82 ± 1.0 × 10^7^
		10–12 cmbsf	<0.08	26.2	0.03	544.01	<0.05		2.64 ± 0.1 × 10^8^	6.71 ± 0.6 × 10^7^
Outside active seeps area	PC11	3–4 cmbsf	<0.08	8.09	<0.03	412.92	<0.05	ND	1.63 ± 0.06 × 10^8^	2.70 ± 0.3 × 10^7^
		10–12 cmbsf	<0.08	26.94	<0.03	544.19	<0.05		5.98 ± 0.06 × 10^7^	1.76 ± 0.1 × 10^7^
	PC12	3–4 cmbsf	<0.08	13.03	<0.03	440	<0.05	ND	1.91 ± 0.01 × 10^8^	3.45 ± 0.3 × 10^7^
		10–12 cmbsf	<0.08	27.27	<0.03	561.32	<0.05		8.63 ± 0.9 × 10^7^	2.00 ± 0.1 × 10^7^

### Microbial abundance in the Gulf of Mexico sediments

Microbial abundances, estimated using quantitative PCR, were different in the Gulf of Mexico sediments from seep and non-seep sites (Table 1). In the sediments outside the seepage areas (PC12 and PC11), 16S rRNA gene abundance (bacterial plus archaeal) was 1.5 ± 0.6 × 10^8^ 16S rRNA genes.g^−1^ throughout the sediments cores. In these sediments, bacterial 16S rRNA genes represented 82% of the total 16S rRNA gene abundance with 1.25 ± 0.6 × 10^8^ 16S rRNA genes.g^−1^. In Site 1 sediments (PC5 and PC6), total 16S rRNA gene abundance was up to 10 times higher than in the non-seep sediments with an average of 9.7 ± 2.1 × 10^8^ 16S rRNA genes.g^−1^ in the 3–4 cmbsf sediment layer of PC5 and throughout PC6. Microbial abundance increased in the bottom (10–12 cmbsf) sediment layer of PC5 which contained 3.13 × 10^9^ 16S rRNA genes.g^−1^. In both PC5 and PC6 sediment cores, bacteria were predominant in the 3–4 cmbsf sediment layer (85% of the total 16S rRNA gene abundance) whereas a similar amount of *Bacteria* and *Archaea* was detected in the 10–12 cmbsf sediments layers. In Site 2 sediments (PC9 and PC10), microbial abundances were heterogeneous probably due to extremely localized seepage. In PC9 sediment core, 3.6 × 10^8^ 16S rRNA genes.g^−1^ were quantified and bacterial genes represented 80% of the total 16S rRNA gene abundance. By contrast, more than 1.3 × 10^9^ 16S rRNA genes.g^−1^ were detected throughout the PC10 sediment core with the archaeal genes representing 52% and 57% of the prokaryote total 16S rRNA genes at 3–4 cmbsf and 10–12 cmbsf respectively.

### Microbial community composition in the Gulf of Mexico sediments

**Figure 1:**
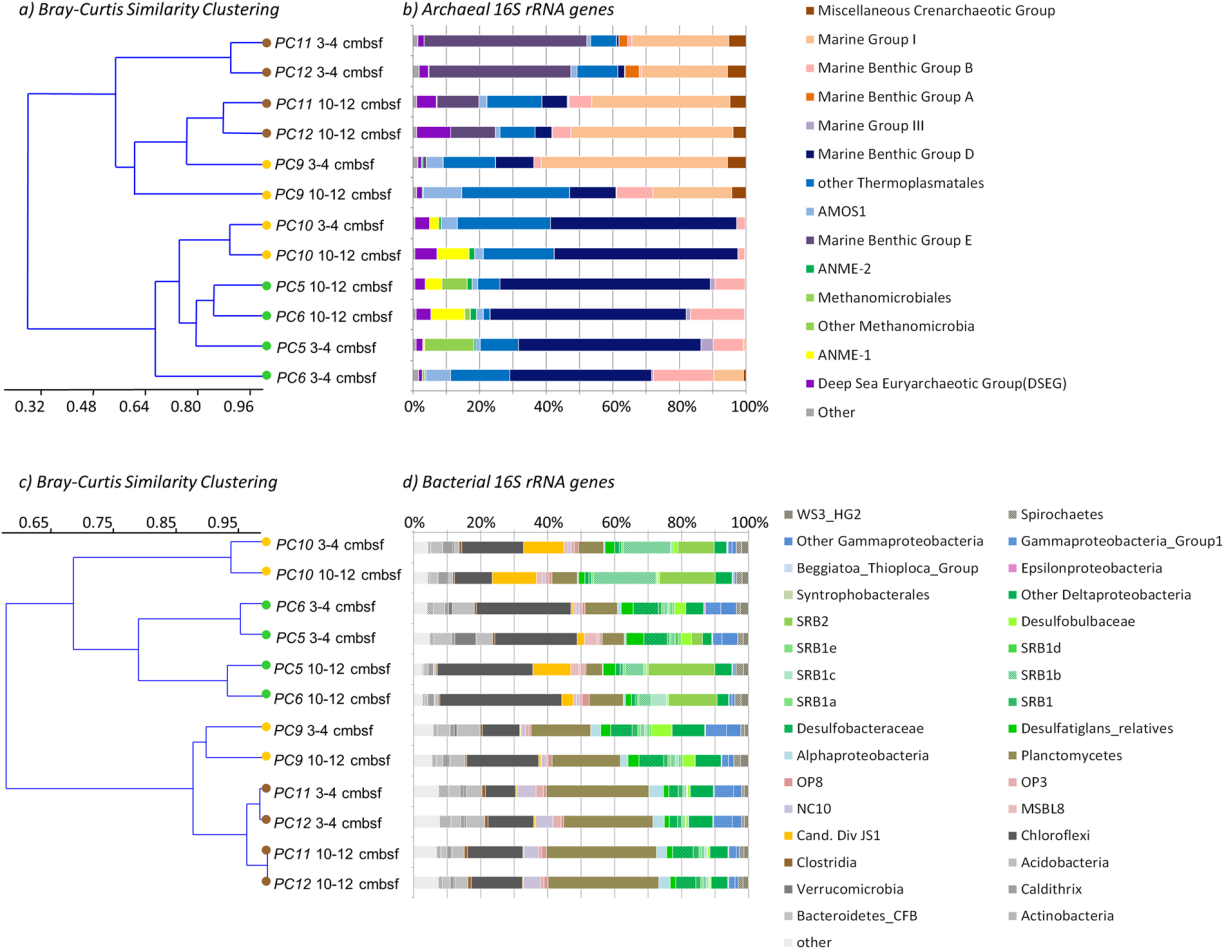
16S rRNA gene amplicons analysis. (**a**) Bray-Curtis similarity clustering of the samples based on (**b**) archaeal 16S rRNA gene sequences (OTU at 97% similarity). (**c**) Bray-Curtis similarity clustering of the sample based on (**d**) bacterial 16S rRNA gene sequences (OTU at 97% similarity). Samples not associated with active seeps (PC11 and PC12) are labeled with brown dots, Site1 oil seep samples (PC5 and PC6) with green dots and Site2 oil seep samples (PC9 and PC10) with yellow dots.

Microbial community composition of the Gulf of Mexico sediments was determined by 16S rRNA gene sequencing with an average of 9.41 ± 5 × 10^4^ reads per sample. Both archaeal and bacterial sequence analyses indicated a similar clustering of the samples (Fig. 1a and c). Sediments samples from outside the seep area (PC11 and PC12) presented a community composition that was distinct from the seep communities (One-Way NPMANOVA *F:*23.66*p:*0.0023, SIMPER average dissimilarity: 49.5), with the exception of the PC9 sediment core that exhibited a geochemical and microbial abundance profile similar to sediments from outside the seep area (Table 1). Excluding PC9 samples, SIMPER analysis of the archaeal 16S rRNA gene dataset indicated that Marine Benthic Group E (45 ± 4% of the archaeal reads at 3–4 cmbsf in PC11 and PC12) and Marine Group I were predominant in the non-seep samples (44 ± 5% of the archaeal reads at 10–12 cmbsf in PC11 and PC12) and explained together up to 37% of the dissimilarity between archaeal community composition in sediments from seeps compared to sediments from outside seep areas (Fig. 1b). Similarly, SIMPER analysis of the bacterial 16S rRNA gene dataset indicated that members of the *Planctomycetes* were predominant in the non-seep samples (28 ± 3% of the bacterial reads throughout PC11 and PC12) and explained up to 19% of the dissimilarity between the seep samples and those from outside the seep areas. The archaeal community of the seep samples was characterized by dominance of members of Marine Benthic Group D (55 ± 7% of the sequences, 30% of the dissimilarity; SIMPER) and the presence of ANME-1 at 10–12 cmbsf (8 ± 2% of the sequences, 4% of the dissimilarity; SIMPER) (Fig. 1b). Bacterial communities in the seep samples contrasted with samples from outside the seep area due to the presence of several deltaproteobacterial lineages, notably the SEEP SRB2 group, closely related to the *Syntrophobacteraceae* (14 ± 4% of the bacterial 16S rRNA gene sequence reads in PC10 and at 10–12 cmbsf in PC5 and PC6), and the SEEP SRB1b affiliated to the *Desulfosarcina*/*Desulfococcus* group (*Desulfobacteraceae*) (Fig. 1d). Candidate Division JS1 (*Atribacteria*) related sequences were also identified only in the seep sediment samples. JS1 sequences represented a large proportion of the reads at 10–12 cmbsf (9 ± 4% of the bacterial 16S rRNA gene reads in PC10, PC5 and PC6 at 10–12 cmbsf). Additionally, differences between seep samples (PC10 vs PC5 and PC6) were observed. A larger proportion of *Chloroflexi* (28 ± 4% of the sequences, 20% dissimilarity; SIMPER) was detected in Site 1 (PC5 and PC6) whereas larger proportions of SEEP SRB1b and SEEP SRB2 related sequences (15 ± 3 and 12.5 ± 4% of the sequences respectively, 18 and 11% of the dissimilarity; SIMPER) were observed in PC10 (Fig. 1d).

Community composition of methanogens/anaerobic methanotrophs and sulfate-reducing bacteria was also investigated by high throughput sequencing of methyl coenzyme M reductase alpha subunit genes (*mcrA*) and dissimilatory sulfite reductase (*dsrAB*) genes (Supplementary Figure 1). No methanogens or ANME were detected in the sediments outside areas with active seepage whereas ANME1 and ANME2 related *mcrA* genes as well as various methanogens-related sequences were detected in all seep samples (Supplementary Figure 1b). Sulfate-reducing bacteria were identified in all samples. Substantial proportions of reads from members of the *Desulfobacteraceae* (62 ± 4% of the *dsrAB* reads) were detected in PC10 sediment samples whereas a larger proportions of reads from the *Syntrophobacteraceae* (28 ± 6% of the *dsrAB* sequences) were identified in samples from outside seep areas (Supplementary Figure 1d). *Desulfatiglans anilini* (*Desulfobacteraceae*)-related sequences (13 ± 9% of the *dsrAB* sequences) were also detected at higher relative abundance in seep samples (Supplementary Figure 1d). Additionally, a significant proportion of sequences detected in all samples were affiliated to environmental clusters 9, 10 and 13 as defined by Muller *et al*. (2015), which lack any known cultured representative (Supplementary Figure 1d).

### Comparative Metagenomic Analysis of the Gulf of Mexico sediments

Given the high similarity between PC5 and PC6 as well as between PC11 and PC12, metagenome analysis was conducted on seep sediment samples from PC5 and PC12 representative of seep site 1 and the non seep area respectively. PC10 sediment core was selected as representative of the seep site 2 since PC9 exhibited a geochemical and microbial abundance profile similar to sediments from outside the seep area. Shotgun metagenome sequencing was carried out on 6 sediment samples (3–4 and 10–12 cmbsf sediment layers from PC5, PC10 and PC12). Despite numerous attempts using Metabat^41^ and GroopM^42^, metagenomic binning of draft genomes was unsuccessful. This is probably due to high variability of the sequences coupled to limited sequencing depth, which make assembly challenging^43^. Therefore our metagenomic analysis focused on comparative distribution of reads between the three sites.

**Figure 2:**
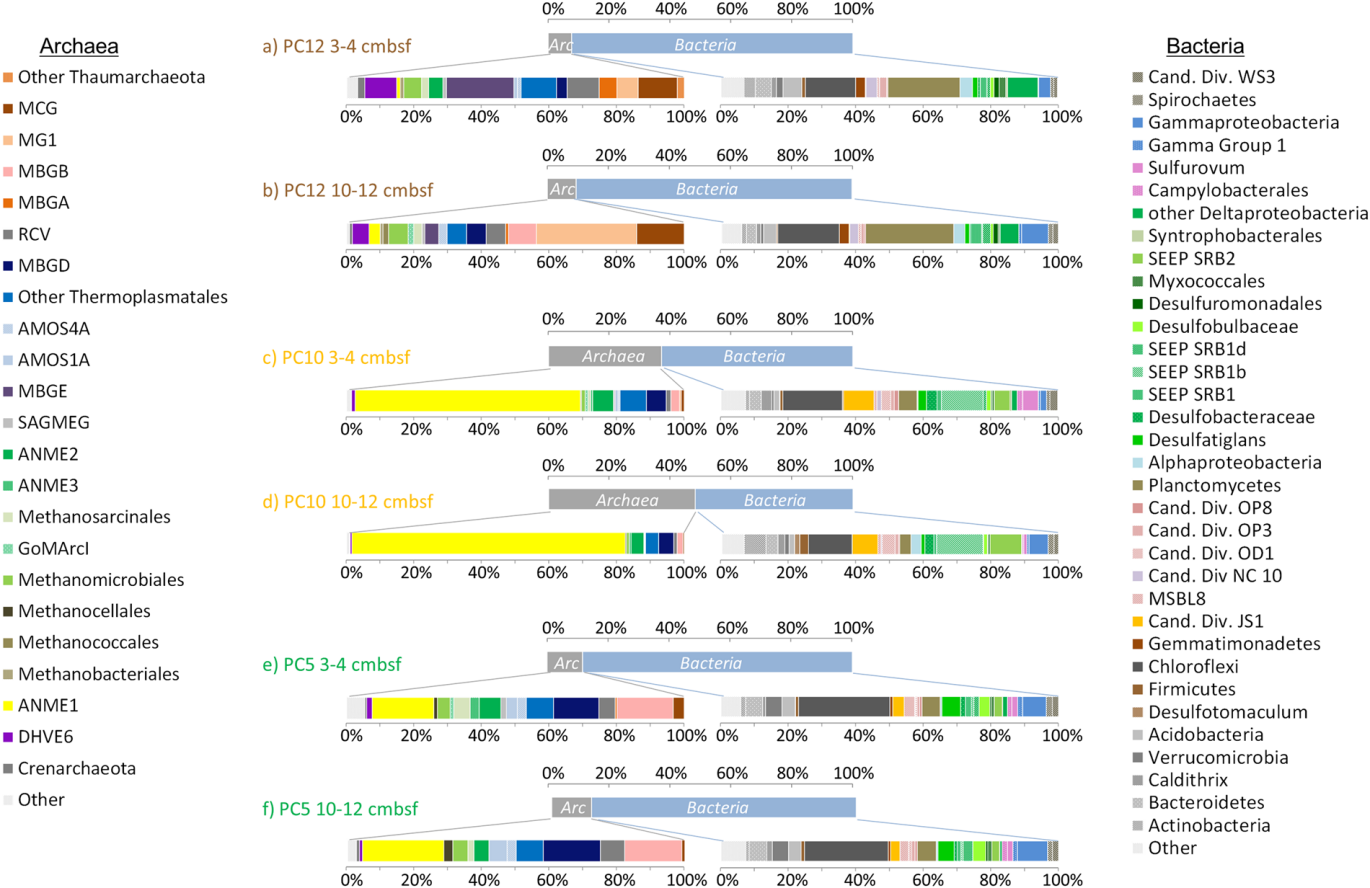
Relative proportion and affiliation of 16S rRNA genes recovered from shotun metagenomes in (**a**) 3–4 cmbsf and (**b**) 10–12 cmbsf sediment layers of PC12 (non seep sample); (**c**) 3–4 cmbsf and (**d**) 10–12 cmbsf sediment layers of PC10 (Site 2 methane seep); (**e**)3–4 cmbsf and (**f**) 10–12 cmbsf sediment layers of PC5 (Site 1 oil seep).

16S rRNA gene sequences were extracted from the metagenomes. The relative proportions of different microbial groups was similar to estimates from qPCR with 90% of the 16S rRNA reads affiliated to bacteria in the sediments outside the seep area and up to 46% of the 16S rRNA gene reads from seep site 2 (PC10) affiliated with *Archaea* (Fig. 2). Although, taxonomic affiliations of the bacterial 16S rRNA reads were consistent with the 16S rRNA gene amplicon libraries (correlation *r* = 0.86, *p* < 0.01), archaeal diversity obtained by shotgun metagenomics presented a different picture, notably for the PC10 sample (correlation r = 0.14, p = 0.05). In the metagenome data ANME-1 16S rRNA genes represented up to 80% and 14% of the archaeal 16S rRNA reads in PC10 and PC5 sediments respectively (Fig. 2). Which contrast with the lower relative abundance of ANME-1 reads in amplicon based analyses (<10%; Fig. 1b). This discrepancy is probably due to primer selectivity since the ANME-1 16S rRNA gene sequences recovered from metagenomes had 3 mismatches with the primers used in amplicon-based analyses. Consequently, the relative proportion of members of the MBGD as well as *Thermoplasmatales*, detected as predominant archaeal members of the archaeal community by 16S rRNA gene amplicon analysis (Fig. 1b), represented less than 5 and 10% of reads recovered from PC10 and PC5 metagenome data respectively.

**Figure 3:**
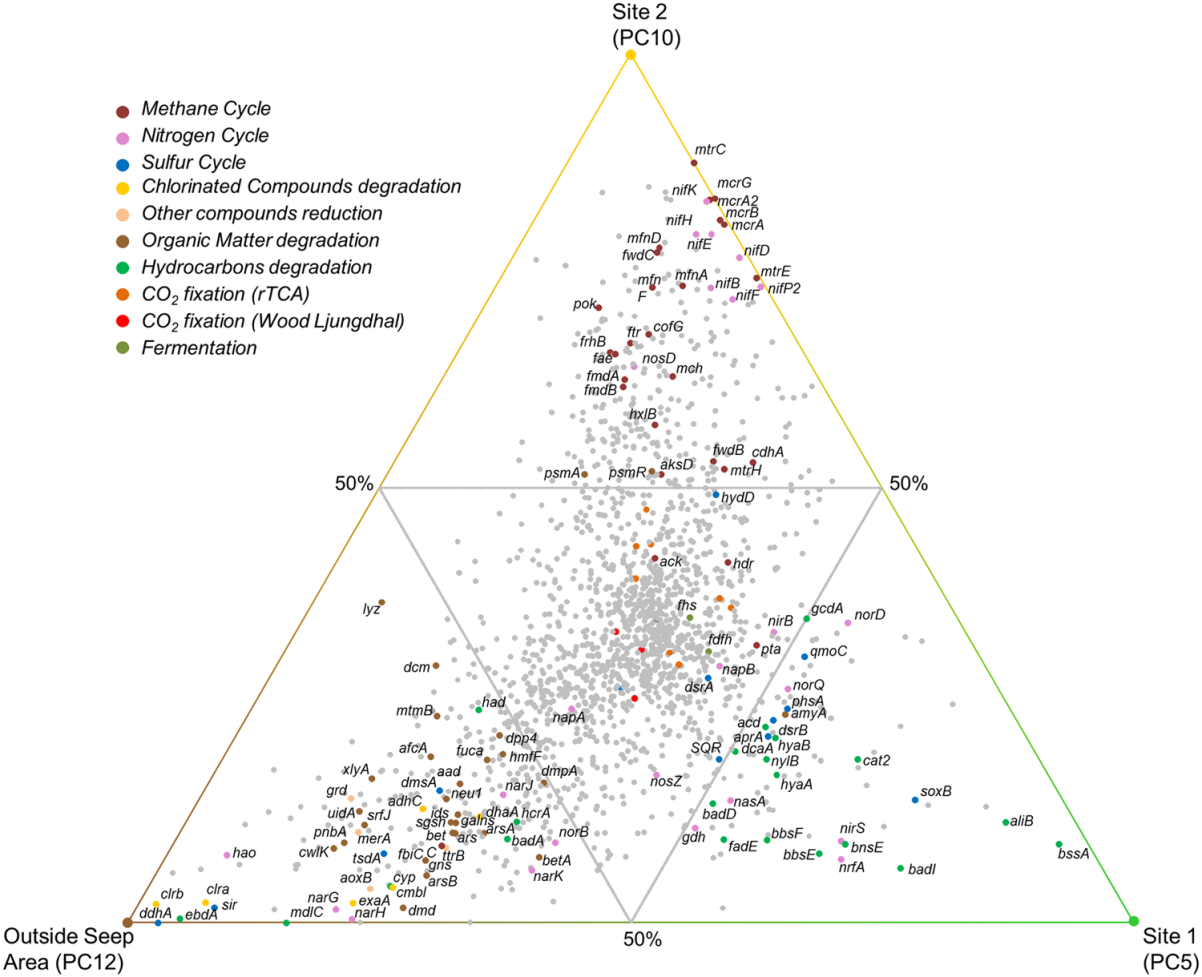
Ternary plot of genes identified in PC12 (non seep, brown corner), PC10 (Site 2 methane seep, yellow corner) and PC5 (Site 1 oil seep, green corner) sediment cores. Each dot represents a single gene. 1795 genes were represented, corresponding to the genes with the most differential representation between the sediment cores and together explaining 80% of the dissimilarity between metagenomes after normalization. The colors of the dots correspond to specific metabolic pathways. The closer the symbol is to the node of the triplot, the more predominant the gene is in that particular sediment site compared to the others. Genes in the center are shared among all three sites. Owing to the large number of shared genes between studied sites only genes overrepresented in one sample (more than 50% of all the genes found in 3 cores are found in one specific core) were investigated. In addition, specific pathways were also analyzed. A list of identified genes with a description and KEGG orthology is provided in Supplementary Table 3.

**Figure 4:**
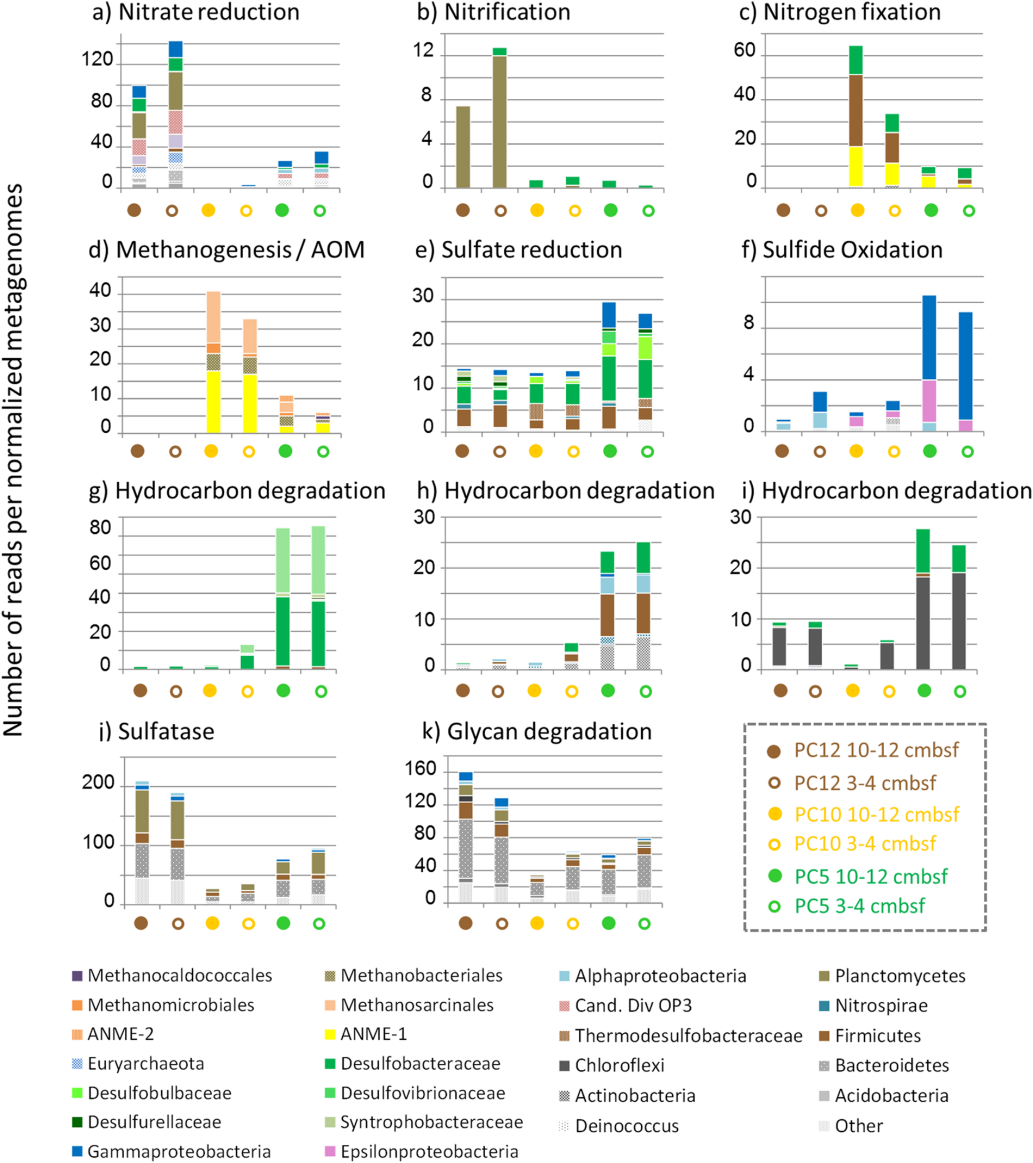
Relative proportion and taxonomic affiliation of specific genes in each normalized metagenome for selected metabolic processes. (**a**) Nitrate reduction (*narG*); (**b**) Nitrification (*hao*); (**c**) Nitrogen fixation (*nifK*); (**d**) Methanogenesis/anaerobic oxidation of methane (*mcrA*); (**e**) Sulfate reduction (*dsrB*); (**f**) Sulfide oxidation (*SoxB*); (**g**) Hydrocarbon degradation (*bssA*); (**h**) Hydrocarbon degradation (*aliB*); (**i**) Hydrocarbon degradation (*bnsE*); (**j**) sulfatase (*aslA*); (**k**) Glycan degradation (*fuca*).

No significant difference in metabolic potential was observed between samples from 3–4 and 10–12 cmbsf (Fig. 2; total metagenome: Bray-Curtis Similarity between 3–4 and 10–12 cmbsf > 90%), suggesting conserved metabolic capabilities throughout each sediment core. Therefore metagenomes from the 3–4 and 10–12 cmbsf sediment layers were considered as representative for each core in the metagenomic comparative analysis. Ternary plot of the metabolic potential identified from metagenome analysis of the three sediment cores highlighted different metabolic capabilities between the three sites (Fig. 3). The microbial community from samples outside the seep area (PC12) had an overrepresentation of genes involved in nitrogen cycling (*narGH*, *hao*), chlorinated compound degradation (*clrAB*, *exaA*, *adhC*), breakdown of sulfonated heteropolysaccharides (*aslA*, *arsAB*, *gns*, *galns*, *betC*, *ids*) and degradation of other polysaccharides and sugars (*neu*, *fucA*, *srfJ*, *uidA*). Based on the taxonomic signature of these genes, nitrogen cycle genes (25% of *narG* reads and 95% of *hao* reads) were mainly affiliated with marine *Planctomycetes* (*Candidatus Scalindua*) (van de Vossenberg *et al*., 2013), whereas the genetic potential for the initial breakdown of sulfonated compounds and polysaccharides were identified in members of the *Planctomycetes* (31%), *Bacteroidetes* (31%) and *Firmicutes* (13%) (Fig. 4). By contrast, a high representation (70–80% of all genes) of nitrogen fixation (*nifBEFHK*) and anaerobic methane oxidation pathways (*mcrABCG*, *mtrEC*, *mfnADF*, *fmdABC*) were detected in the sediment core from the methane-dominated site 2 seep sample PC10 (Fig. 3). Genes for nitrogen fixation were mainly affiliated to *Firmicutes* (45%), ANME-1 (30%) and *Desulfobacteraceae* (24%), whereas genes involved in AOM were mainly affiliated with the ANME-1 lineage (47%) (Fig. 4). Likewise, various genes involved in hydrocarbon degradation (*bssA*, *bssEF*, *bnsE*, *hyaAB*, *badDI*, *aliB*) as well as genes from the sulfate reduction pathway (*dsrB*, *aprA*) were over represented in sediment samples from seep site 1 (PC5) which contained more complex hydrocarbons (Fig. 3). Sulfate reduction genes were mainly affiliated to members of *Deltaproteobacteria* (57%) and *Firmicutes* (17%). Taxonomic affiliations of hydrocarbon degradation genes indicated potential for toluene degradation (*bssA*) in *Deltaproteobacteria* (*Desulfobacteraceae* (45%) and *Syntrophobacteraceae* (47%)), cyclohexane degradation in *Actinobacteria* (23%), *Firmicutes* (34%), *Alpha*- (14%) and *Deltaproteobacteria* (*Desulfobacteraceae 22%*) and polycyclic aromatic hydrocarbons degradation (*bnsE*) in *Deltaproteobacteria* (*Desulfobacteraceae* 27%) and *Chloroflexi* (72%) (Fig. 4).

## Discussion

The Gulf of Mexico exhibits high sedimentation rates (1–10 mm.y^−1^) and the accumulation, burial and maturation of this organic matter over geological timescales has generated widespread natural hydrocarbon seep environments such as cold seeps, gas hydrates, brines and asphalt volcanoes^44^. Geochemical analysis of the sampled seep areas indicated that the seep fluids were distinct with respect to their composition. In Site 1 (PC5 and PC6) methane and more complex hydrocarbons (UCM, aromatic compounds and hopanoids) were detected in the sediments, as previously observed in thermogenic gas hydrates and seep of the Mississippi Canyon^40^ whereas methane was the only hydrocarbon identified in Site 2 sediments, which is consistent with methane hydrates and seep also observed in the Canyon^40^. Therefore, in this study, microbial community composition and metabolic potential among three contrasting environments in the Gulf of Mexico were compared: a methane seep (PC9 and PC10), a hydrocarbon seep (PC5 and PC6) and sediments from outside the seep areas (PC11 and PC12). Potential for CO_2_ fixation via the reverse tricarboxylic acid cycle and the Wood Ljungdahl pathway as well as the potential for fermentation (formate and acetate) were highly represented in all samples (Fig. 3). This is consistent with the preponderance of organotrophic and chemosynthetic lifestyles in deep marine sediments.

### Sediments with no evidence of active hydrocarbon seepage

Although non-seep areas represent the vast majority of all marine sediments, they remain relatively poorly investigated compared to seep sediments. Methane concentration was extremely low (<0.05 µM) in the non-seep sediments analyzed. Moreover, no *mcrA* genes were detected in metagenomes or by targeted amplification. This is consistent with the geochemical data that indicated that these sampling sites were not influenced by seepage of hydrocarbons. 16S rRNA gene analyses (both amplicon-based and metagenomic) indicated a specific community composition with an overrepresentation of the *Planctomycetes*, Marine Group I and Marine Benthic Group E archaea as previously observed in seafloor sediments from the Guaymas basin, with minimal influence of hydrocarbon seepage^14,22^. Comparative analysis of the metagenomes indicated an overrepresentation of several metabolic genes, notably affiliated to the *Planctomycetes* and *Bacteroidetes*, involved in the degradation of various glycans as well as chlorinated and sulfonated compounds, as previously detected by fosmids sequencing^45^. In marine environments, chlorinated compounds such as chloromethane and other chloroalkanes are derived from phytoplankton and algae (Gribble, 2003), while sulfonated compounds have been found as a major constituent of humic acids and algae (agar and fucans)^46^. Likewise glycans are abundant polysaccharides in marine invertebrate tissues and algae^45^. The communities in the non-seep site were therefore likely adapted to grow on detrital organic matter delivered to the sediment as marine snow and decaying organic matter particles. As has been previously observed^22^ it was not possible to extract RNA of sufficient quality or quantity for subsequent analysis, from the non-seep sediments (data not shown). This observation, as well as the frequent detection of Marine Group I archaea and *Planctomycetes* in the water column^47^ and/or attached to macroscopic detrital aggregates^48^, suggest that these microbes may predominantly have a pelagic lifestyle, degrading the heteropolysaccharides of the marine snow, and primarily represent organisms deposited in the sediment with water column-derived organic matter and have limited activity *in situ* in these marine sediments. Nitrate reduction potential, inferred from *narG* genes was detected in members of *Planctomycetes*, candidate division OP3, *Euryarchaeota* and *Proteobacteria*. However, metabolic activity of these bacteria and archaea might be limited in marine sediments by the low availability of nitrate (below detection limit in all sediment samples) and nitrite. Sulfate-reducers were detected by targeted amplifications (16S rRNA and dsrAB genes) but were represented by a minority of reads in shotgun metagenome data, and sulfate was only depleted in the upper sediment layer. If sulfate reduction is not balanced by the considerable potential for sulfate generation from sulfonated organic compounds (a high proportion of sulfatase genes were identified in the metagenome data) or sulfide oxidation by *Alpha*- and *Gammaproteobacteria* detected in the sediments, these results suggest that the activity of the sulfate reducers detected in these sediments, is limited. Thus it is likely that the ecosystem in non-seep sediments is driven largely by utilization of water column-derived organic carbon, and that this is insufficient to support complete reduction of seawater derived sulfate. The corollary of this is that sulfate-reduction is not supported by upwelling sources of organic carbon, consistent with the lack of evidence of hydrocarbon seepage in these sediments. The detection of sulfate at seawater levels at 10–12 cmbsf in these sediments suggests that the surface sediment sulfate-reducing communities may to some extend be fed by seawater advection into the sediments but that even then the organic matter delivered to the sediments from the water column, is insufficient to support complete sulfate reduction.

### Hydrocarbon seeps versus methane seeps

Cold seeps are defined by the upward advection of methane and other hydrocarbons from the subsurface seabed to the seafloor^49^. Previous global analysis of 16S rRNA genes recovered from seafloor ecosystems identified a specific microbiome in cold seeps environments^30^, however distinctions between methane and oil seep microbial community and metabolic profiles have received much less attention^11^. Multigenic (16S rRNA, *dsrAB* and *mcrA* gene) and metagenome sequencing highlighted a qualitatively similar microbial community composition between the methane and oil seeps. Archaeal (ANME-1, ANME-2, MBGD and *Thermoplasmatales*) and bacterial (*Desulfobacteraceae*, *Syntrophobacteraceae*, *Chloroflexi*, candidate division JS1) lineages were present as had previously been noted in Gulf of Mexico cold seep sediments^5,11^, supporting the concept of a characteristic seep microbiome^30^. However, shotgun metagenomic sequencing highlighted different relative proportions of these lineages depending on the nature of the seep fluids. A large proportion of ANME-1 (up to 37% of total 16S rRNA reads) was detected in methane seep sediments, as previously observed^11^. In line with this observation, the complete anaerobic methane oxidation pathway (reverse methanogenesis without the *mer* gene), previously identified in ANME-1^50^, was overrepresented in metagenomes from these sediments (Fig. 3) indicating considerable potential for anaerobic methane oxidation. Relative abundance of ANME-1 was highly correlated with SEEP SRB1B (*r* = 0.94 *p* = 0.004) and SEEP SRB2 (*r* = 0.89, *P* = 0.01), consistent with findings in other anoxic cold seep sediments^30^. However, these SRB are not known as direct syntrophic partners for the anaerobic oxidation of methane by ANME-1 in cold sediments. Furthermore, the representation of ANME-1 16S rRNA genes in the metagenome dataset (14 and 80% in PC5 and PC10 respectively) was disproportionate with any potential syntrophic bacteria (0.8% of 16S rRNA reads affiliated to SEEP SRB1a or *Desulfobulbus*). Therefore these results could suggest that ANME-1 in our methane seep sediments are likely to oxidize methane by the reverse methanogenesis pathway in a bacterial syntrophy-independent process, as previously proposed^50,51^. The particular abundance of ANME-1 in the methane rich seeps would suggest that anaerobic methane oxidation was the predominant microbial process in these seeps.

By contrast, a larger proportion of *Desulfobulbaceae*, *Syntrophobacteraceae*, *Desulfatiglans* and *Chloroflexi* were identified in the hydrocarbon seep sediments, where residual oil and aromatic hydrocarbons were observed. Gene markers for anaerobic degradation of aromatic hydrocarbons (*bssA*, *aliB*, *badI*, *bbsE*) were overrepresented in these sediments, suggesting that in the presence of other hydrocarbons along with methane, the metabolic potential of the microbial community may be based on breakdown of more complex hydrocarbons rather than on methane oxidation. The hydrocarbon degradation genes were mainly affiliated to members of the *Desulfobacteraceae* and *Syntrophobacteraceae*, as previously observed by a *masD/assA/bssA* amplicon survey of cold seep sediments^31^. 16S rRNA genes of from *Desufatiglans*, which are related to cultivated species known to degrade alkyl-substitued and unsubsituted aromatic hydrocarbons^32^, and other sub-groups of the *Desulfobacteraceae* (SEEP SRB1d) were overrepresented in sediment samples affected by seepage of hydrocarbons other than methane. It is therefore likely that these uncultured *Deltaproteobacteria* may degrade aromatic hydrocarbons, as previously suggested^26,34^ and contribute to the UCM, characteristic of biodegraded oil, detected in these sediments. An overrepresentation of sulfate reduction genes (*aprA*, *dsrB*) from *Desulfobacteraceae*, and pore water sulfate depletion were observed in these samples, as in other oily seeps^52^. Therefore, energy for hydrocarbon degradation in marine seepages is likely provided by sulfate reduction, supporting previous activity measurements in Gulf of Mexico seep sediments that suggested sulfate reduction activity was fuelled by non-methane hydrocarbons^11,52^. An overrepresentation of sulfide oxidation genes (*Sox*), from known mat-forming *Gamma*- and *Epsilonproteobacteria* (*Beggiatoa* and *Sulfurovum* spp.) was also apparent in the metagenome data from these sediments (Fig. 4), suggesting that microbial mat formation may also rely on these hydrocarbon-degrading sulfate-reducers. *Firmicutes (Clostridia)*-affiliated genes for aromatic hydrocarbon degradation and sulfate reduction were also detected in the metagenomic dataset, though they represented a minority in 16S rRNA and *dsrAB* gene surveys. Members of the *Firmicutes* phylum such as *Clostridia* and *Desulfotomaculum* are known oil degraders^33^, therefore they may also contribute to the degradation of hydrocarbon in these seep sediments. Furthermore, the potential for polycyclic aromatic hydrocarbon (naphthalene) degradation (*bnsE*) was also identified in members of the *Chloroflexi* phylum. *Chloroflexi* have been frequently detected in marine sediments^39,53^ and have been implicated in a number of metabolic processes (dechlorination^54^, fatty acid degradation^39^, aromatic compound degradation and sulfate reduction^55^) depending on the particular *Chloroflexi* lineage. BLAST analysis of full length 16S rRNA sequences reconstructed from metagenomic data (data not shown) indicated that *Chloroflexi* sequences were affiliated to *Anaerolinaceae* and closely related to *Chloroflexi* sequences previously amplified from mud volcanoes^56^. *Anaerolinaceae* have been identified as abundant components of anaerobic hydrocarbon-degrading enrichments^57–59^. Together, this suggests that considerable metabolic variability occurs within the *Chloroflexi* phylum and some cold seep-associated *Anaerolinaceae* lineages may be hitherto unknown aromatic hydrocarbon degraders. Consistent with the UCM detected by GC analysis, the metabolic potential of the resident microbial community appears to be adapted to the composition of the seepage with different catabolic pathways for the degradation of aromatic hydrocarbons being present.

Due to the prevalence of seep environments, Gulf of Mexico sediments represent a unique opportunity to observe the influence of seep hydrocarbon composition on microbial community structure and function. Although, the microbial community analysis presented here supports the concept of a characteristic seep microbiome, our sampling strategy and comparative metagenomic approaches indicated different microbial communities and metabolic traits depending on the composition of the seep fluids. Therefore, even if these metabolic capacities remain to be confirmed by larger scale investigation, activity measurements or transcriptomic analyses, they indicate that seafloor ecosystems can be sustained by different microbial activities depending on percolating fluid composition.

## Experimental Procedures

### Sample description

Sediments push core samples were collected in October 2015 at three different locations in the Mississippi Canyon of the Gulf of Mexico using an ROV. Duplicate push cores were taken approximatively 20 cm apart at each sampling site. Site 1 (PC5 and 6) at 1055 meters water depth, was covered by a thin microbial mat and had unambiguous geochemical evidence of seepage. Site 2 (PC9 and 10), located approximatively 70 meters away, at 1129 meters water depth had scattered white mat-like traces at the sediment surface. Site 2 was identified by seafloor acoustic reflectivity but no fluid emission were observed on the seafloor. Finally two sediment push-cores (PC11 and 12) were taken 200 meters away at locations remote from any visible active seepage area (1128 meters water depth) as ‘background’ reference samples.

Sediment cores were recovered to the research vessel and immediately sectioned aseptically. Sediment layers from 1–3, 5–10 and 12–15 centimeters below the seafloor (cmbsf) were subsampled for oil and gas analysis whereas samples from 3–4 and 10–12 cmbsf were subsampled for microbiology and porewater analysis. Samples for microbiology were immediately stored in 50 ml sterile tubes at −80 °C until nucleic acid extractions could be performed. Nucleic acids (DNA and RNA) were extracted from 5 grams of sediments as previously described^60^ and re-suspended in 200 µl of DNA-, RNA-, RNase- free water. Extracted nucleic acids were purified using a Wizard DNA clean-up kit (Promega, Madison, WI, USA) and were stored at −20 °C until further analysis. During nucleic acid extraction, visible oil was present in the 11–12 cmbsf sample from PC5 and PC6.

Methane concentration and hydrocarbon composition in sediment samples were determined by gas chromatography and GC MSxMS (Shimadzu, Kyoto, Japan). After nucleic acid extraction, pore water was collected from all the samples following centrifugation. Anions were quantified using a Dionex ICS-2000 ion chromatograph with suppressed conductivity detection. Anions were separated on an AS11 column using a KOH gradient.

### Quantitative Polymerase Chain Reaction (qPCR) analysis

Abundance of *Bacteria* and *Archaea* in the Gulf of Mexico sediments was estimated using quantitative (q)PCR targeting 16S rRNA genes with primers Bact1369f/Bact1492r and Arc787f/Arc1059r respectively (Supplementary Table 1). Quantification was performed in triplicate with different template concentrations (0.5, 1, 1.5 ng of DNA) to detect any PCR inhibition. Amplification reactions were carried out in a Rotor-Gene Q system (Qiagen, Hilden, Germany) in a final volume of 25 µl using Brilliant III superMix (Agilent, Santa Clara, CA, USA), 0.5 µM of each primers and 5 µl of DNA template. qPCR conditions were as follows: 40 cycles of denaturation at 95 °C for 15S then annealing and extension at 60 °C for 60S. Standard curves were prepared in triplicate with dilutions ranging from 0.001 to 100 nM of DNA extracted from *Desulfobulbus propionicus* (ATCC 33891) and *Methanococcoides methylutens* (ATCC 33938). The R^2^ of standard curves obtained by qPCR were above 0.99 and PCR efficiencies above 94%. qPCR results were expressed in terms of 16S rRNA gene copies per gram of sediments.

### Illumina Miseq Amplicon library preparation, sequencing and analysis

Microbial community composition of the Gulf of Mexico sediments was determined by high throughput 16S rRNA gene sequencing. The V4-V5 region of bacterial 16S rRNA genes (420 bp product) was amplified using primers S-D-Bact-0516-a-S-18/S-D-Bact-0907-a-A-20 whereas the V1-V2-V3 region of archaeal 16S rRNA genes (530 bp product) was amplified using primers S-D-Arch-0008-b-S-18/S-D-Arch-0519-a-A-19 (Supplementary Table 1) as previously applied in hydrocarbon rich marine sediments^14^. Additionally, sulfate reducers and methanogens were investigated by amplification and sequencing of the dissimilatory (bi)sulfite reductase (*dsrAB*) gene using primers DSR1728/DSR4R (380 bp product) and the methyl coenzyme M reductase (*mcrA*) gene using primers MLf/MLr (550 bp product) (Supplementary Table 1). All these primer sets produce PCR products that allow pair-end sequences to be assembled. Miseq adaptors (Supplementary Table 1) were fused to the 5′ region of the primers. All PCR reactions were conducted in triplicate with negative controls using Brilliant III super Mix (Agilent), 1 µM of each primers and 1 µl of DNA template in a 25 µl reaction. All PCR assays comprised 30 cycles of denaturation at 95 °C for 30 s, annealing for 30 s at the appropriate temperature (58 °C for 16S rRNA genes, 55 °C for *dsrAB* gene, 50 °C for *mcrA* gene) and extension for 30 s at 72 °C followed by a final extension step at 72 °C for 5 min. Replicate amplicons were pooled and purified from agarose gels using a Qiagen MinElute Gel purification kit (Qiagen, Hilden, Germany). PCR products were indexed using a Nextera XT kit (Illumina Inc., San Diego, CA, USA) according to manufacturer’s recommendations. Indexed amplicons were quantified using a Qubit dsDNA HS Assay Kit (Life Technology, Carlsbad, CA, USA) and diluted to give an equimolar mix of products at a final concentration of 4 nM for Miseq library preparation. DNA libraries were diluted to 4 pM then pair-end Illumina MiSeq sequencing was performed using an Illumina Miseq v3 kit (illumina Inc., San Diego, CA, USA), as recommended by the manufacturer. This generated 2 × 300 bp pair-end sequences. Datasets were split into reads from individual indexed amplicons *in silico* using Miseq reporter software. Reads were assembled into single pair-end sequences which were curated using Qiime^61^. OTU picking was carried out using the *de novo* OTU picking option. Sequences with low quality scores or flagged as chimeras using UChime were removed. Alignment and determination of the taxonomic affiliation of the reads were carried out using BLAST against Silva release 119^62^, *dsrAB* ^63^ and *mcrA* ^64^ sequences databases as references. Raw sequences were deposited in the NCBI public database under Bioproject PRJNA385797.

### Metagenomic library preparation sequencing and analysis

Metagenomes for 6 sediment samples were constructed from 1 ng of metagenomic DNA using a Nextera XT Library Kit (Illumina, San Diego, CA, USA) according to manufacturer’s recommendations. Tagmentation and indexing were checked using a High Sensitivity DNA chip on an Agilent Bioanalyzer 2100 (Agilent Technologies, Santa Clara, CA, USA). Metagenomes were normalized and diluted to 4 nM based on the average DNA fragment size and concentration determined from the Bioanalyzer analysis. Two metagenomes were pooled in equimolar quantities for each sequencing run. Metagenome libraries were diluted to 14.3 pM and sequenced using an Illumina Miseq V3 kit (Illumina, San Diego, CA, USA). Barcode and adapter sequences were removed from the metagenome data on-instrument using Illumina’s MiSeq Reporter software and the sequence data were exported as fastq files. Datasets were quality filtered using Trimmomatic^65^, using default setting for paired-end Illumina data. Paired-end joining was done using the ‘join_paired_ends.py’ script bundled with the QIIME package (version 1.9), using default settings. Assembly was performed from paired-end joined reads and unpaired reads passing quality filtering using MEGAHIT^66^, using default settings. After assembly, all reads which passed quality filtering where mapped back to the assembled contigs to detect reads which were not included in the assembly, using BBMap^67^, with default settings. Reads which were not mapped to the assembly were concatenated with assembled contigs into a single fasta file for upload to the IMG/M analysis pipeline^68^ for gene calling and functional annotation. For each sample, coverage and mapping data file (BAM file) was also uploaded to IMG/M pipeline to preserve relative abundance of the genes. Metagenomes were normalized by rarefaction for sample comparison. Metagenome data are available in IMG/M under the following accession numbers: 3300008340, 3300008410, 3300008416, 3300008417, 3300008465 and 3300008468 (detail in Supplementary Table 2). 16S rRNA gene sequences were extracted from the metagenome data using vsearch^69^ against the same Silva database used for amplicon analysis (Silva release 119). Reconstruction of full length 16S rRNA genes was done using EMIRGE^70^.

### Statistical analysis

Statistical analyses (correlation tables, One-Way NPMANOVA, SIMPER) were performed using PAST software^71^ according to the guide for statistical analysis in microbial ecology^72^. For amplicon dataset, distance matrices between samples were determined on 97% operational taxonomic units using the Bray-Curtis dissimilarity index. Differences in community composition and metabolic potential (Number of identified Kegg orthologies) were tested using One-way NPMANOVA and SIMPER tests based on Bray-Curtis dissimilarity measures.

## Electronic supplementary material

Supplementary Material
