# Supplementary material for: Comparative metagenomics of hydrocarbon and methane seeps of the Gulf of Mexico
Source: Sci Rep. 2017 Nov 22;7:16015. doi: 10.1038/s41598-017-16375-5 (PMC5700182; doi:10.1038/s41598-017-16375-5)
Supplement: Supplementary file 1 — Supplementary Material [file 41598_2017_16375_MOESM1_ESM.pdf]

Comparative metagenomics of hydrocarbon and methane seeps of the Gulf of Mexico

Adrien Vigneron[1,2], Eric B. Alsop[2,3], Perrine Cruaud[4], Gwenaelle Philibert[2], Benjamin King[2], Leslie Baksmaty[2], David Lavallée[2], Bartholomeus Lomans[5], Nikos C. Kyrpides[3], Ian M. Head[1] and Nicolas Tsesmetzis[2]*

Supplementary Figure 1: *mcrA* and *dsrB* gene amplicons analysis. a) Bray-Curtis similarity clustering of the sample based on b) *mcrA* gene sequences. c) Bray-Curtis similarity clustering of the sample based on d) *dsrB* gene sequences. Samples not associated with active seeps (PC11 and PC12) are labeled with brown dots, Site 1 oil seep samples (PC5 and PC6) with green dots and Site 2 oil seep samples (PC9 and PC10) with yellow dots.

Supplementary Table 1: Primer sets used in this study

Supplementary Table 2: Description of the metagenomes

Supplementary Table 3: List of identified genes in the ternary plot with description and Kegg orthology

Supplementary Figure 1 : *mcrA* and *dsrB* gene amplicons analysis

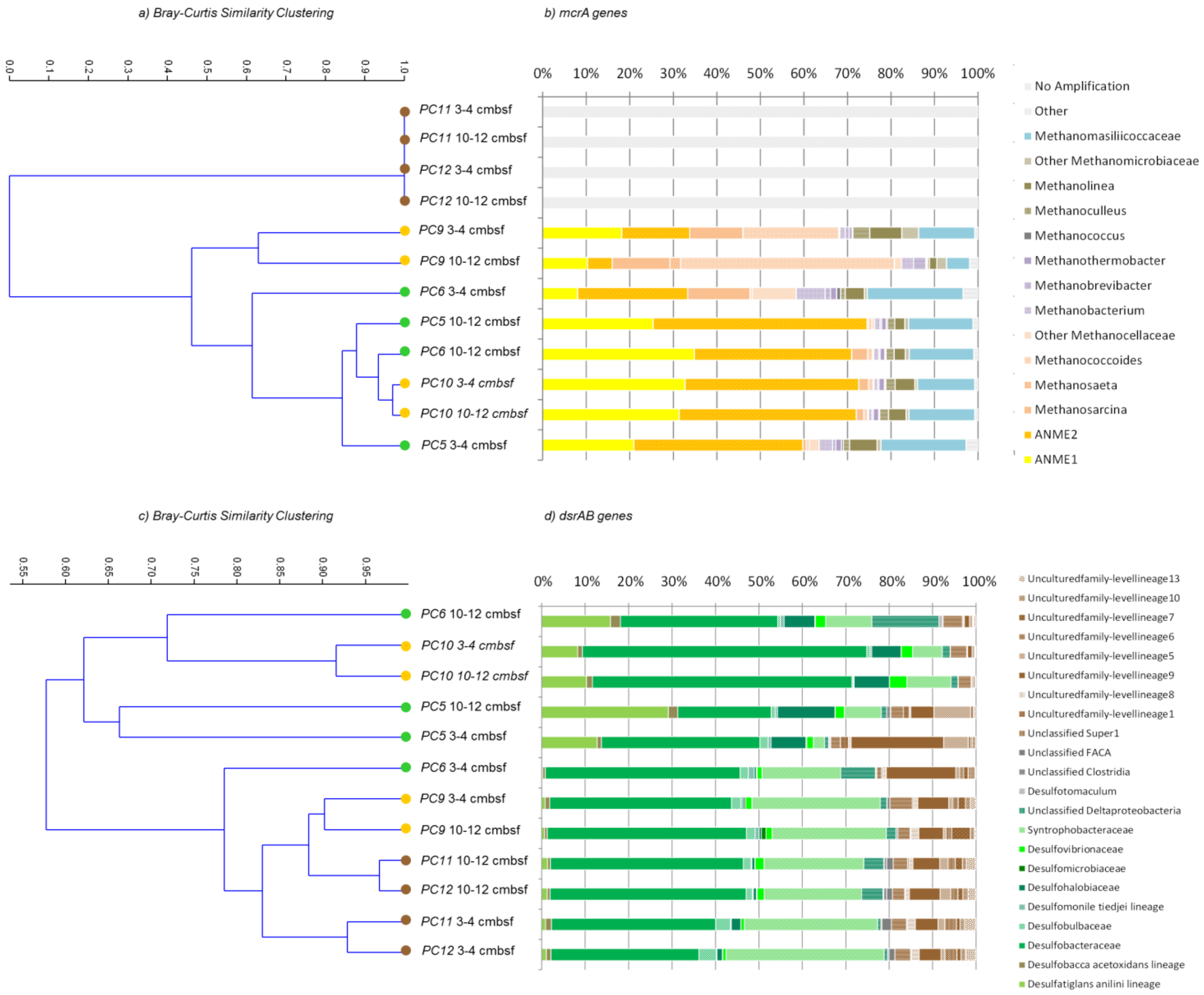

Supplementary Table 1 : Primer sets used in this study

Primer Name	Function	Target Group	Sequence (5’-3’)	Amplicon size	Annealing temp. (°C)	Primer Conc. (µM)	Ref.
BACT1369F<br>BACT1492R	Q-PCR	*Bacteria*	CGG-TGA-ATA-CGT-TCY-CGG<br>GGW-TAC-CTT-GTT-ACG-ACT-T	142	60	0.6	Suzuki et al., 2000
ARC787F<br>ARC1059R	Q-PCR	*Archaea*	ATT-AGA-TAC-CCS-BGT-AGT-CC<br>GCC-ATG-CAC-CWC-CTC-T	273	60	0.5	Yu et al., 2005
S-D-Bact-0516-a-S-18<br>S-D-Bact-0907-a-A-20	Sequencing	*Bacteria*	TGC-CAG-CAG-CCG-CGG-TAA<br>CCG-TCA-ATT-CMT-TTG-AGT-TT	420	58	0.5	Dufresne et al. 1996 / Yu and Morrison 2004
S-D-Arch-0008-b-S-18<br>S-D-Arch-0519-a-A-19	Sequencing	*Archaea*	TCY-GGT-TGA-TCC-TGS-CGG<br>GGT-DTT-ACC-GCG-GCK-GCT-G	530	58	0.5	Fish et al. 2002 / Sorensen and Teske 2006
DSR1728Fmix<br>Dsr4Rmix	Sequencing	*dsrB*	CAY-ACC-AGG-NTG-G and variants<br>CAG-TTA-CCG-CAG-TAC-AT and variants	360	55	0.5	Muller et al. 2015
MLf<br>MLr	Sequencing	*mcrA*	GGT-GGT-GTM-GGA-TTC-ACA-CAR-TAY-GCW-ACA-GC<br>TTC-ATT-GCR-TAG-TTW-GGR-TAG-TT	550	55	0.5	Luton et al. 2002
Adaptor F<br>Adaptor R	Sequencing	-	TCGTCGGCAGCGTCAGATGTGTATAAGAGACAG<br>GTCTCGTGGGCTCGGAGATGTGTATAAGAGACAG	-	-	-	-

Supplementary Table 2 : Description of the metagenomes

IMG ID	Sample ID	Study Site	Sample Description	Assembly method	Total Size (bp)	Nbr Reads	%GC	Nbr Contigs	Total Lenght Contig (bp)	Gene count	rRNA gene Count	Total KO count
**3300008416**	MPC12B	Outside seeps area	PC12 10-12 cmbsf	IDBA_UD	1177881052	4601097	48.57	551236	331901444	4458807	16125	901599
**3300008417**	MPC12T	Outside seeps area	PC12 3-4 cmbsf	IDBA_UD	902097346	3922162	48.97	511286	298566422	3522118	11256	780281
**3300008465**	MPC5B	Site 1 – hydrocarbons seeps	PC5 10-12 cmbsf	IDBA_UD	225578278	1025355	48.08	53685	36687802	875359	3271	267651
**3300008410**	MPC5T	Site 1 – hydrocarbons seeps	PC5 3-4 cmbsf	IDBA_UD	270318699	1201416	49.18	66604	47501469	1064509	3770	325883
**3300008340**	MPC10B	Site 2 – Methane seeps	PC10 10-12cmbsf	IDBA_UD	310096569	1348245	45.17	235179	152890251	1029736	3992	252670
**3300009874**	MPC10T	Site 2 – Methane seeps	PC10 3-4 cmbsf	IDBA_UD	422857366	1838510	45.99	206387	144163483	1470248	5465	363406

Supplementary Table 3 : List of identified genes in the ternary plot with description and Kegg orthology

Methane Cycle

gene	Kegg Orthology	Enzyme name
mtrC	k00579	tetrahydromethanopterin S-methyltransferase subunit C
mcrG	k00402	methyl-coenzyme M reductase gamma subunit
mcrA2	K00400	methyl coenzyme M reductase system
mcrB	K00401	methyl-coenzyme M reductase beta subunit
mcrA	K00399	methyl-coenzyme M reductase alpha subunit
mtrE	k00581	tetrahydromethanopterin S-methyltransferase subunit E
mfnD	k06914	tyramine---L-glutamate ligase
fwdC	k00202	formylmethanofuran dehydrogenase subunit C
mfnF	k07072	(4-(4-[2-(gamma-L-glutamylamino)ethyl]phenoxymethyl)furan-2-yl)methanamine synthase
pok	k06982	pantoate kinase
cofG	k11780	FO synthase subunit
ftr	K00672	formylmethanofuran--tetrahydromethanopterin N-formyltransferase
fae	k10713	5,6,7,8-tetrahydromethanopterin hydro-lyase
frhB	k00441	coenzyme F420 hydrogenase
mch	K01499	methenyltetrahydromethanopterin cyclohydrolase
fmdA	k00200	formylmethanofuran dehydrogenase subunit A

fmdB	k00201	formylmethanofuran dehydrogenase subunit B
hxlB	k08094	6-phospho-3-hexuloisomerase
aksD	k16792	homoaconitase
mtrH	k00584	tetrahydromethanopterin S-methyltransferase subunit H
cdhA	k00192	acetyl-CoA decarbonylase/synthase
pta	K13788	phosphate acetyltransferase
fbiC	k11779	FO synthase
ack	K00925	acetate kinase
hdr	k03388	Coenzyme B-Coenzyme M heterodisulfide reductase

Nitrogen Cycle

nifK	k02591	nitrogenase molybdenum-iron protein beta chain
nifH	K02588	nitrogenase iron protein
nifE	k02587	nitrogenase molybdenum-cofactor
nifD	K02586	nitrogenase molybdenum-iron protein alpha chain
nifB	k02585	nitrogenase FeMo cofactor biosynthesis protein
nifF	k03839	nitrogenase
nifP2	k02589	nitrogenase promotor
nosD	k07218	nitrous oxidase accessory protein
nirB	K00362	nitrite reductase (NADH) large subunit
norD	k02448	nitric oxide reductase
napB	K02558	nitrate reductase cytochrome C550 subunit
norQ	k04748	nitric oxide reductase
nasA	k00372	assimilatory nitrate reductase catalytic subunit
gdh	k15371	glutamate dehydrogenase
nosZ	K00376	nitrous-oxide reductase
napA	K02557	periplasmic nitrate reductase
nirS	k15864	nitrite reductase (NO-forming) / hydroxylamine reductase
nrfA	k03385	nitrite reductase (cytochrome c-552)
narG	K00370	nitrate reductase / nitrite oxidoreductase

narH	K00371	nitrate reductase / nitrite oxidoreductase, beta subunit
narK	k02575	nitrate/nitrite transporter
norB	K04561	nitric oxide reductase subunit B
narJ	K00374	nitrate reductase delta subunit
hao	k10535	hydroxylamine dehydrogenase

Sulfur Cycle

hydD	K17996	sulfhydrogenase subunit delta
qmoC	K16887	quinone membrane oxidoreductase
dsrA	K11180	sulfite reductase alpha subunit
dsrB	K11181	sulfite reductase beta subunit
sat	K00958	sulfate adenylyltransferase
SQR	K17218	sulfide:quinone oxidoreductase
aprA	K00394	adenylylsulfate reductase
phsA	K08352	thiosulfate reductase / polysulfide reductase chain A
soxB	K17224	sulfur-oxidizing protein SoxB
tsdA	k19713	thiosulfate dehydrogenase
sir	k00392	sulfite reductase
dmsA	k07306	dimethylsulfoxide reductase
ddhA	k16964	dimethylsulfide dehydrogenase subunit alpha

Other respiration

aoxB	k08356	arsenate reductase
merA	k00520	mercuric reductase
grd	k10672	betaine reductase
ttrB	k08353	tetrathionate reductase

Hydrocarbon Degradation

bssA	k07540	benzylsuccinate synthase, alpha subunit
aliB	k04117	cyclohexanecarboxyl-CoA dehydrogenase

badA	k04110	benzoate-CoA ligase
badD	k04112	benzoyl-CoA reductase subunit C
badI	k07536	2-ketocyclohexanecarboxyl-CoA hydrolase
bbsE	k07543	benzylsuccinate CoA-transferase
bbsF	k07544	benzylsuccinate CoA-transferase
fadE	k06445	acyl-CoA dehydrogenase
hyaA	k06282	hydrogenase small subunit
hyaB	k06281	hydrogenase-1 large chain
nylB	k01453	6-aminohexanoate-oligomer exohydrolase
dcaA	k06446	acyl-CoA dehydrogenase
acd	k00249	acyl-CoA dehydrogenase
cat2	k18122	4-hydroxybutyrate CoA-transferase
gcdA	k01615	glutaconyl-CoA decarboxylase
bnsE	k15569	naphthyl-2-methylsuccinate CoA transferase subunit
hcrA	k04107	4-hydroxybenzoyl-CoA reductase subunit alpha
mdlC	k01576	phenylglyoxylate carboxy-lyase
had	k07538	6-hydroxycyclohex-1-ene-1-carbonyl-CoA dehydrogenase

Chlorinated Compound degradation

adhC	k00121	S-(hydroxymethyl)glutathione dehydrogenase
dhaA	k01563	haloalkane dehalogenase
cmbl	k01061	carboxymethylenebutenolidase
exaA	k00114	alcohol dehydrogenase
clrA	k17050	perchlorate reductase
clrB	k17051	perchlorate reductase

Organic Matter Degradation

psmA	k03432	Proteasome
psmR	k03420	Proteasome

amyA	k01176	alpha-amylase
lyz	k13915	lysozyme C
dcm	k00558	(cytosine-5)-methyltransferase 1
mtmB	k16176	methylamine---corrinoid protein Co-methyltransferase
dpp4	k01278	Dipeptidyl-peptidases
hmfF	k16874	2,5-furandicarboxylate decarboxylase 1
fuca	k01206	alpha-L-fucosidase
afcA	k15923	alpha-L-fucosidase 2
xlyA	k01447	N-acetylmuramoyl-L-alanine amidase
aad	k04072	acetaldehyde dehydrogenase
neu1	k01186	sialidase-1
galns	k01132	N-acetylgalactosamine-6-sulfatase
ids	k01136	iduronate 2-sulfatase
sghs	k01131	steryl-sulfatase
betC	k01133	choline-sulfatase
betA	k00108	Choline deshydrogenase
ars	k01138	arylsulfatase
arsA	k01134	arylsulfatase A
arsB	k01135	arylsulfatase B
dmpA	k01266	aminopeptidase
gns	k01137	N-acetylglucosamine-6-sulfatase
srfJ	k01201	glucosylceramidase
uidA	k01195	beta-glucuronidase
pnbA	k03929	para-nitrobenzyl esterase
